# The First Genetic Map in Sweet Osmanthus (*Osmanthus fragrans* Lour.) Using Specific Locus Amplified Fragment Sequencing

**DOI:** 10.3389/fpls.2017.01621

**Published:** 2017-09-22

**Authors:** Yanxia He, Wangjun Yuan, Meifang Dong, Yuanji Han, Fude Shang

**Affiliations:** ^1^Plant Germplasm Resources and Genetic Laboratory, College of Life Sciences, Henan University Kaifeng, China; ^2^Institute of Pharmacy, Pharmaceutical College of Henan University Kaifeng, China; ^3^Woe Key Laboratory of Plant Stress Biology, Henan University Kaifeng, China

**Keywords:** genetic map, pseudo-testcross, SLAF-seq, SNP, *Osmanthus fragrans*

## Abstract

*Osmanthus fragrans* is an ornamental plant of substantial commercial value, and no genetic linkage maps of this species have previously been reported. Specific-locus amplified fragment sequencing (SLAF-seq) is a recently developed technology that allows massive single nucleotide polymorphisms (SNPs) to be identified and high-resolution genotyping. In our current research, we generated the first genetic map of *O. fragrans* using SLAF-seq, which is composed with 206.92 M paired-end reads and 173,537 SLAF markers. Among total 90,715 polymorphic SLAF markers, 15,317 polymorphic SLAFs could be used for genetic map construction. The integrated map contained 14,189 high quality SLAFs that were grouped in 23 genetic linkage groups, with a total length of 2962.46 cM and an average distance of 0.21 cM between two adjacent markers. In addition, 23,664 SNPs were identified from the mapped markers. As far as we know, this is the first of the genetic map of *O. fragrans*. Our results are further demonstrate that SLAF-seq is a very effective method for developing markers and constructing high-density linkage maps. The SNP markers and the genetic map reported in this study should be valuable resource in future research.

## Introduction

Sweet osmanthus (*Osmanthus fragrans* Lour. 2*n* = 46) is a woody, evergreen shrub or small tree species in the *Oleaceae* family. It is widely distributed and owns cultural significance in China, and was introduced into Europe in the late eighteenth century (Zang et al., 2003). The genus *Osmanthus* has been categorized into 27 species, 24 species of which are distributed in China, and including the most representative species of *O. fragrans*. A total of 166 cultivars have been identified since China acquired International Cultivar Registration Authority (ICRA) status for *O. fragrans* cultivars in 2004. *O. fragrans* cultivars are divided into four groups depending on their flowering season and corolla coloration. Cultivars of the Asiaticus Group, Albus Group and Luteus Group bloom only in autumn, while cultivars of the Aurantiacus Group can flower for several times in different seasons except summer (Xiang and Liu, 2007). The maintenance and reproduction of the cultivars are accomplished through cuttage.

Cultivated for a long time in China, *O. fragrans* is one of the important commercial flowers and a traditional horticultural plant. So it has been widely cultivated due to its unique fragrance, aesthetic and cultural values (Shang et al., 2003; Yuan et al., 2011). Products derived from this plant, such as food, tea, beverages, perfumes, and cosmetics, are widely appreciated (Wang et al., 2009; Wu et al., 2009; Baldermann et al., 2010). The medicinal value of *O. fragrans* has long been recognized in traditional Chinese medicine according to the Compendium of Materia Medica (Xiang and Liu, 2007). Therefore, this plant is very popular among people and has been extensively researched.

Genetic study in *O. fragrans* achieved great progress. As an androdioecious species (Hao et al., 2011; Xu et al., 2014), the pollination and breeding systems of *O. fragrans* cultivars is complex, with selfing and crossing coexisting in the populations (Li, 2014). The karyotype of this species was reported as 2*n* = 2*x* = 46 (Li et al., 2004). With the development of the next-generation sequencing technology (NGS), several housekeeping genes, carotenogenic genes implied in corolla coloration, and other genes associated with fragrance and pigment biosynthesis were identified based on transcriptome dataset (Mu et al., 2014, 2017; Zhang et al., 2016). However, it still remains unknown about the genetic and molecular mechanisms underlying diverse biological traits. So far, little has been reported on the segregation analysis of genes or the molecular markers in a cross-population. Therefore, a genetic linkage map has become a vital tool for identifying functional genes (Graham et al., 2010), facilitating genome analysis (Takehisa et al., 2012), and elucidating the genetic basis of a trait of interest (Ward et al., 2013). Up to now, many genetic linkage maps have been reported for some tree species, including *Eucommia ulmoides* (Wang et al., 2014), *Betula platyphylla* (Jiang et al., 2011), *Eucalyptus* sp. (Freeman et al., 2006), and *Populus* sp. (Cervera et al., 2001). However, no a genetic map of *O. fragrans* has been developed until now. The availability of molecular markers is prerequisite for constructing a high-density linkage map, and the lack of these is one of the main reasons that hinder the development of genetic maps in many species.

Great efforts have been made on revealing genetic diversity of *O. fragrans* through using different molecular markers. High levels of genetic diversity among *O. fragrans* cultivars were detected by AFLP and RAPD markers (Shang et al., 2004; Yuan et al., 2011). And a similar result was demonstrated in the 139 individuals from natural populations by microsatellite (SSR) markers (Hu et al., 2014). However, the available information for these markers is insufficient to build the genetic map of *O. fragrans*. Single nucleotide polymorphism (SNP), a mainstream molecular marker, was identified as the most suitable for construction of a high-resolution genetic linkage map due to the abundance and ubiquity of SNPs in living organisms (Wang et al., 2009). The emergence of NGS technology makes it possible to quickly exploit large numbers of SNP in the genomic DNA. Specific-locus amplified fragment sequencing **(**SLAF-seq), an approach basing on NGS, was recently reported (Sun et al., 2013). This method can complete large-scale *de novo* SNP discovery and genotyping in a single process with a high-resolution strategy. Additionally, the use of an enhanced reduced representation library (RRL) sequencing technique enable greater specificity and accuracy of SLAF-seq compared to other NGS-based methods, such as complexity reduction of polymorphic sequences (CRoPS) (Van Orsouw et al., 2007), restriction site-associated DNA sequencing (RAD-seq) (Baird et al., 2008), and genotyping-by-sequencing (GBS) (Elshire et al., 2011). SLAF-seq has therefore become a preferred method for developing high-density genetic maps for species in the absence of a reference to genome sequences. Several high-density genetic maps have been successfully created using this method over the past 2 years, such as *Agropyron cristatum* (Zhang et al., 2015), *Camellia sinensis* (Ma et al., 2015), *Prunus mume* (Zhang et al., 2015), *Paeonia* sect. *Moutan* (Cai et al., 2015), and *Juglans regia* (Zhu et al., 2015).

The objective of this study is to construct the first genetic map for *O. fragrans* by the use of the SLAF-seq method. The framework genetic map is the initial step in understanding the genome organization of this important species, and a well-defined genetic map would further facilitate the cloning of trait related genes and QTL (quantitative trait locus) fine mapping of some important traits in *O. fragrans*.

## Materials and methods

### Plant material and genomic DNA extractions

Two cultivars, “Wan Yingui” (Albus Group) and “Huangchuan Jingui” (Luteus Group), were selected to be the hybrid parents. “Wan Yingui” is a 25 years old male cultivar. “Huangchuan Jingui” is a 20 years old hermaphrodite cultivar and was employed as female parent. So the bagging and emasculation are necessary in the process of artificial pollination. The parents showed obvious differences in gender, corolla color and leaf traits. The hybridization was performed at the Garden of Huangchuan Jingui (32°04′11.71″ N, 115°04′10.92″ E), Huangchuan County, Henan Province, in the fall of 2014, and the fruits were harvested in the spring of 2015. The seeds would usually take 1–2 years to germinate normally. To shorten this period, the hybrid seeds were subjected to in vitro embryo culturing according to Wangjun et al. (2005) at the Germplasm Resources and Genetic Engineering Laboratory of the College of Life Sciences, Henan University. They all became seedlings within 2 months. The leaves of 129 F1 progenies were collected during their subculturing phase and immediately frozen in liquid nitrogen. These and the leaves of the parent plants were utilized for genomic DNA extraction.

Genomic DNA samples were evaluated using 1% agarose gels and an ND-1000 spectrophotometer (Nano Drop, Wilmington, DE, USA) to ensure it suitable for SLAF-seq in terms of integrity and quality.

### SLAF library construction and high-throughput sequencing

The SLAF library construction and high-throughput sequencing were conducted as the report of Sun et al. (2013) accompanied by a few modifications. The DNA samples of the 129 progenies and two parents were treated with *Rsa*I, ATP, T4 DNA ligase, and Duplex Tag-labeled Sequencing adapters at 37°C. Then the digestion-ligation DNA was used for the subsequent polymerase chain reaction (PCR), together with the essential components of dNTP, Taq DNA polymerase, and primers. The products were then purified, pooled and separated via electrophoresis on 2% agarose gel. The fragments between 314 and 414 bps were extracted and sequenced using the Illumina HiSeq 2500 system (Illumina, Inc.; San Diego, CA, USA) at the Biomarker Technologies Corporation in Beijing.

### SLAF-seq data analysis and genotyping

SLAF marker identification and genotyping were accomplished as following. Low-quality reads in each cycle (quality score <30; a quality score of 30 represents a 0.1% chance of error) were throw away by real-time monitoring during sequencing. Then, those with clear index information were mixed together in view of their sequence similarity, which can be ascertained by BLAT (Kent, 2002). The sequences with above 90% similarity would be grouped and identified as one SLAF marker (locus). The alleles of each SLAF were defined according to their parents, and individuals were genotyped by sequence similarity to their parents (Sun et al., 2013). The SLAFs in this study were classified into three types: Non-Polymorphic SLAF, Polymorphic SLAF, and Repetitive SLAF. As a diploid species, a SLAF can contain at most four genotype tags. Therefore, non-polymorphic SLAFs containing only one tag, together with repetitive SLAFs embracing more than four tags, were ruled out. Only a marker with two, three, or four tags was defined as a polymorphic SLAF and used for further analysis and specific molecular marker development. Polymorphic SLAFs were genotyped according to their population type, which followed eight segregation types (ab × cd, ef × eg, ab × cc, cc × ab, hk × hk, lm × ll, nn × np, and aa × bb). In this study, most Polymorphic SLAFs passed the final filter. Only those that contained <3 SNPs, had an average sequence depth of 10-fold or more in the parents and 2-fold or more in the progenies, and exhibited >70% integrity in mapping population individuals, were picked out for subsequent map construction. The aa × bb type markers, a full sub-type family where the parents are both homozygous, were filtered out.

### Linkage map construction

In the process of genetic mapping, a chi-square test (χ^2^) was carried out to examine the Mendelian segregation ratio for each marker. Markers at the *P* < 0.05 significance level were initially excluded from the core linkage map and separately inserted as accessory markers at a later stage. Map construction was achieved according to the method of HighMap, which was detailed by Liu et al. (2014). Firstly, the markers with high qualities were grouped based on a pair-wise modified logarithm of odds (LOD) score for the recombination frequency. Secondly, those markers were ordered using gibbs sampling, spatial sampling, and a simulated annealing algorithm. At the same time, the map distance was estimated. Thirdly, the incorrect genotypes were recognized and eliminated through the k-nearest neighbor algorithm. The processes of marker ordering and error correcting were carried out iteratively so that the markers could be ordered correctly. Several cycles later, accurate linkage maps were achieved. The map function of Kosambi was used in the process. At last, heat maps and haplotype maps were used to evaluate the map quality.

## Results

### Analysis of SLAF-seq data and SLAF markers

**Figure 1:**
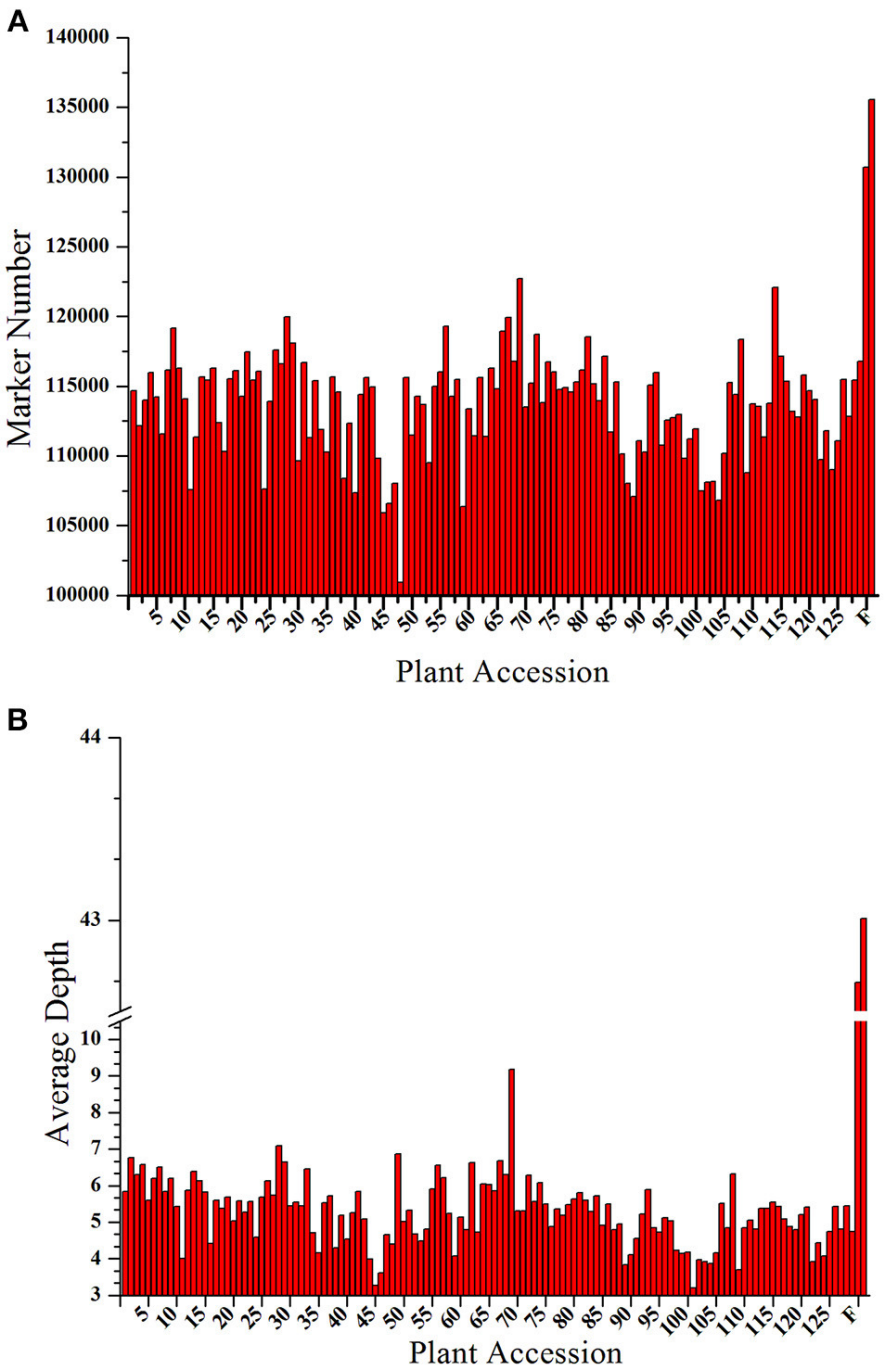
Number and depth of markers for each F1 individual and the two parents. The x-axes for both **(A,B)** indicate the plant accession. The designation consists of the 129 F1 progeny individuals followed by the female parent and the male parent; the y-axes show the number of markers in **(A)** and the marker depth in **(B)** for each sample.

A total of 206.92 M paired-end reads were acquired from the high-throughput sequencing of the constructed SLAF library of both the parents and the 129 progenies. Among these reads, 93.23% of reads had quality scores >Q30, and the guanine cytosine (GC) content was 37.5% on average. Subsequently, 173,537 SLAF markers were derived from the high quality reads, of which 135,540 and 130,698, respectively, were exploited from the female and male parents. The read numbers for SLAFs were 5,575,444 in the female parent and 5,828,967 in the male parent, with the sequencing depth of each SLAF was 43.01- and 42.66-fold, respectively. In the 129 progenies, the reads numbers for the SLAFs varied from 556,112 to 1,592,274, and the development of SLAFs ranged from 100,910 to 122,711 with a marker depth of 5.27-fold on average for each individual (Figure 1). The raw sequence data has been deposited into NCBI SRA with project accession of PRJNA317048.

**Figure 2:**
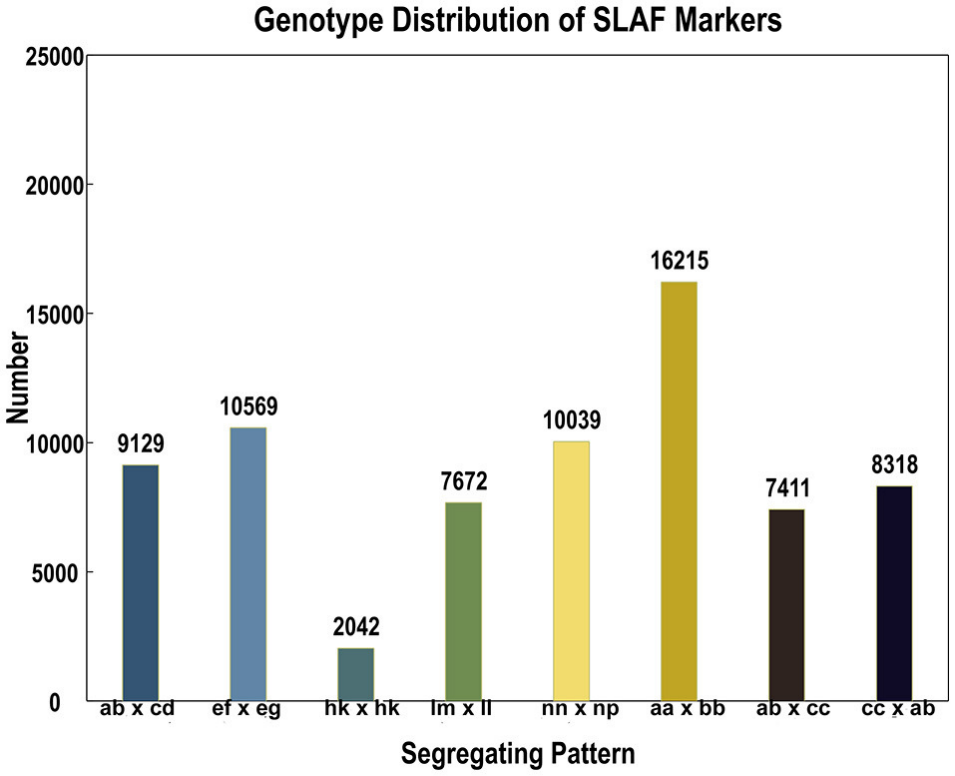
Number of markers in each of eight segregation patterns.

In the 173,537 SLAFs, 52.27% markers were polymorphic, which were used for subsequent genetic genotyping (Table 1). The others, including repetitive SLAFs (0.35%) and non-polymorphism SLAFs (47.38%), were filtered out due to their location in the repeated sequence region or a lack of parent information. Out of all the polymorphic SLAFs, 71,395 markers were successfully genotyped in parents and resulted in eight segregation types according to the genotype encoding rule (Figure 2). The pseudo-testcross theory led to the removal of aa × bb and low-quality markers (the method for removing them is described in section SLAF-seq Data Analysis and Genotyping). This meant that 15,317 polymorphic SLAFs belonging to five segregation patterns (lm × ll, nn × np, hk × hk, ef × eg, and ab × cd) were left for developing the genetic linkage map.

**Table 1:** SLAF mining results.

**Type**	**Polymorphic SLAFs**	**Non-polymorphic SLAFs**	**Repetitive SLAFs**	**Total SLAFs**
Number	90,715	82,214	608	173,537
Percentage	52.27	47.38	0.35	100.00

### Construction of a high-density linkage map

**Figure 3:**
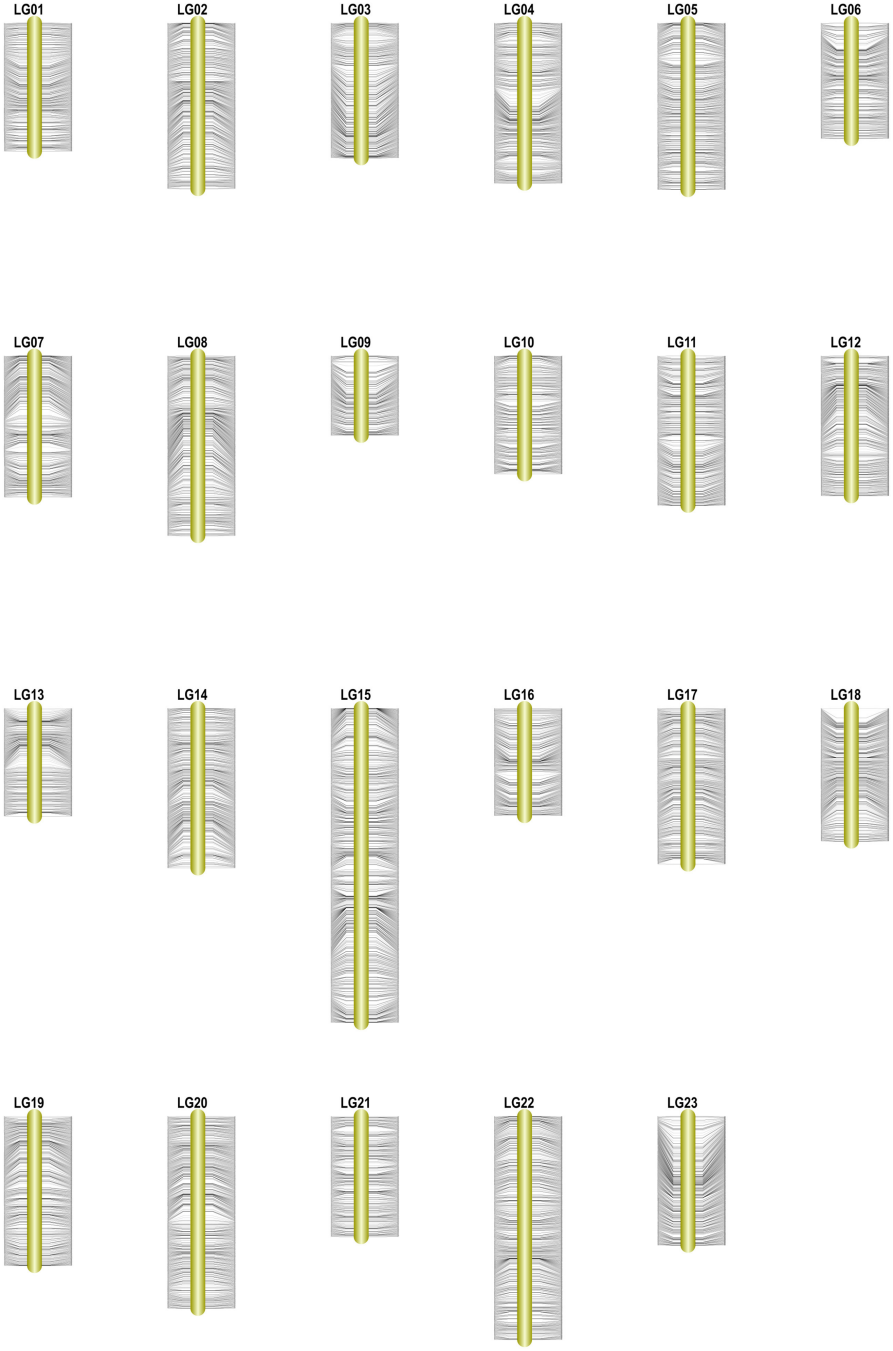
High-density genetic map of *Osmanthus fragrans*. The SLAF markers and their locations are shown on the right and left side, respectively.

Among the 15,317 SLAFs available for the linkage map construction, 14,189 of these were distributed into 23 linkage groups (LGs) according to the modified LOD (MLOD) scores between markers and the linkage analysis. The percentage of SLAF markers available for mapping was about 92.64%, with 9,175 SLAFs available for the female map, 7,196 SLAFs for the male map, and 14,189 SLAFs for the final integrated map (Table 2, Figure 3). The average sequencing depths of the mapped markers were 70.33-fold in the female parent, 69.50-fold in the male parent, and 10.85-fold in the F1 individuals. The total genetic length of the genome was 2952.20 cM for the female map, 2872.91 cM for the male map, and 2962.46 cM for the integrated map. Meanwhile, the average distance between adjacent markers was 0.31, 0.40, and 0.21 cM in each map, respectively.

**Table 2:** Basic characteristics of the 23 linkage groups.

**LG ID**	**Number of markers**			**Size (cM)**			**Average distance between markers (cM)**		
	**Female map**	**Male map**	**Integrated map**	**Female map**	**Male map**	**Integrated map**	**Female map**	**Male map**	**Integrated map**
LG1	357	263	524	122.22	112.57	118.58	0.34	0.43	0.23
LG2	560	250	677	155.79	128.30	143.22	0.28	0.52	0.21
LG3	317	282	551	90.69	96.21	101.94	0.29	0.34	0.19
LG4	374	334	655	93.60	130.97	138.06	0.25	0.39	0.21
LG5	370	356	681	112.54	135.61	138.49	0.30	0.38	0.20
LG6	314	147	472	100.92	104.62	108.08	0.32	0.72	0.23
LG7	405	249	578	149.81	145.29	132.57	0.37	0.59	0.23
LG8	916	419	735	319.50	128.38	124.37	0.35	0.31	0.17
LG9	298	157	325	80.06	117.26	110.69	0.27	0.75	0.34
LG10	423	242	483	131.64	97.29	95.17	0.31	0.40	0.20
LG11	349	352	611	117.57	110.43	102.86	0.34	0.31	0.17
LG12	447	311	572	116.46	111.56	112.44	0.26	0.36	0.20
LG13	376	201	441	107.66	94.67	102.21	0.29	0.47	0.23
LG14	458	319	653	144.36	118.62	134.21	0.32	0.37	0.21
LG15	455	675	1285	141.37	184.81	252.15	0.31	0.27	0.20
LG16	375	245	439	106.76	104.90	92.87	0.29	0.43	0.21
LG17	439	327	637	119.84	132.64	136.15	0.27	0.41	0.21
LG18	462	304	543	119.857	132.89	131.95	0.26	0.44	0.24
LG19	182	265	610	79.84	118.70	118.74	0.44	0.45	0.19
LG20	515	445	785	141.37	174.28	157.82	0.28	0.39	0.20
LG21	334	269	492	83.86	107.50	97.24	0.25	0.40	0.20
LG22	613	483	913	189.07	162.04	175.56	0.31	0.34	0.19
LG23	336	301	527	127.70	123.37	137.09	0.38	0.41	0.26
Total	9,675	7,196	14,189	2952.20	2872.91	2962.46	0.31	0.40	0.21

Table 2 and Figure 3 show that the distribution of markers and the length of the 23 linkage groups were not the same. In the female map, LG8 was the longest (319.50 cM) and had the most markers (916 SLAFs), whereas LG19 was the shortest (79.84 cM) and had the fewest markers (182 SLAFs). In the male and integrated maps, LG15 was the longest (184.81 and 252.15 cM, respectively) and had the most markers (675 and 1,285 SLAFs, respectively). In the male map, LG6 contained the fewest markers (147 SLAFs) while LG13 was the shortest (94.67 cM). In the integrated map, LG9 had the fewest markers (325 SLAFs), whereas LG16 was the shortest (92.87 cM).

The basic characteristics of the markers for the final map are shown in Table 3 and Table S1. On the integrated map, 4,514 and 6,993 SLAF markers were specifically linked to the maternal and paternal progenitors, respectively, and the other 2,682 SLAFs were shared by parents. The results showed that there was a good one to one match of the 23 linkage groups between female and male maps. Most of markers on each linkage group exhibited good synteny (Figures S1–S5), though a few markers site were arranged differently. The female and integrated maps shared 9,675 SLAFs, 10.36% of which exhibited differences in position between two maps, and 669 of 7,196 markers (9.30%) shared by the male and integrated maps showed the different position between them. Out of a total 14,189 SLAFs, 950 showed distorted segregation at the *P* < 0.05 level on the map, which represented a frequency of 6.70%. The distorted markers were distributed unevenly on 19 groups except for LG8, LG16, LG19, and LG21. A total of 185 distorted markers were identified on LG7, which had the highest distorted markers percentage at 19.47%. Correspondingly, 14 of the total 50 segregation distortion regions (SDRs) were also on LG7. The others were scattered on 14 LGs with SDR numbers ranging from 1 to 5.

**Table 3:** Summary of integrated linkage map of *Osmanthus fragrans*.

**LG ID**	**SLAF markers numbers**				**Gaps ≤ 5 (%)**	**Max Gap (cM)**	**Segregation SLAF (*P* < 0.05)**	**Segregation distortion marker percentage**	**Frequency of segregation distortion marker (%)**	**SDR number**
	**Male proprietary**	**Female proprietary**	**Parent shared**	**Total**						
LG1	167	261	96	524	99.43	2.71	108	11.37	20.61	4
LG2	117	427	133	677	99.11	3.47	130	13.68	19.20	3
LG3	175	269	107	551	99.64	3.45	15	1.58	2.72	0
LG4	197	321	137	655	98.32	2.83	34	3.58	5.19	2
LG5	226	325	130	681	97.06	3.63	16	1.68	2.35	1
LG6	97	325	50	472	99.58	4.54	71	7.47	15.04	2
LG7	139	329	110	578	96.53	5.35	185	19.47	32.01	14
LG8	273	316	146	735	99.86	2.41	0	0.00	0.00	0
LG9	143	168	14	325	97.53	8.72	43	4.53	13.23	2
LG10	166	241	76	483	97.10	3.68	44	4.63	9.11	3
LG11	237	259	115	611	99.84	3.22	16	1.68	2.62	1
LG12	202	261	109	572	99.65	3.09	51	5.37	8.92	4
LG13	127	240	74	441	100.00	3.20	36	3.79	8.16	5
LG14	248	334	71	653	98.93	2.82	3	0.32	0.46	0
LG15	369	610	306	1285	95.56	3.76	8	0.84	0.62	0
LG16	141	194	104	439	99.77	3.56	0	0.00	0.00	0
LG17	214	310	113	637	99.69	4.88	29	3.05	4.55	1
LG18	194	239	110	543	99.82	9.35	29	3.05	5.34	2
LG19	163	345	102	610	100.00	3.29	0	0.00	0.00	0
LG20	270	340	175	785	99.87	3.38	61	6.42	7.77	5
LG21	158	223	111	492	100.00	4.14	0	0.00	0.00	0
LG22	300	430	183	913	99.45	3.16	16	1.68	1.75	1
LG23	191	226	110	527	99.43	3.20	55	5.79	10.44	0
Total	4,514	6,993	2,682	14,189	–	–	950	100.00	6.70	50

“*Gap ≤ 5” indicated the percentages of gaps in which the distance between adjacent markers was smaller than 5 cM*.

### Distribution of markers types on the genetic map

**Figure 4:**
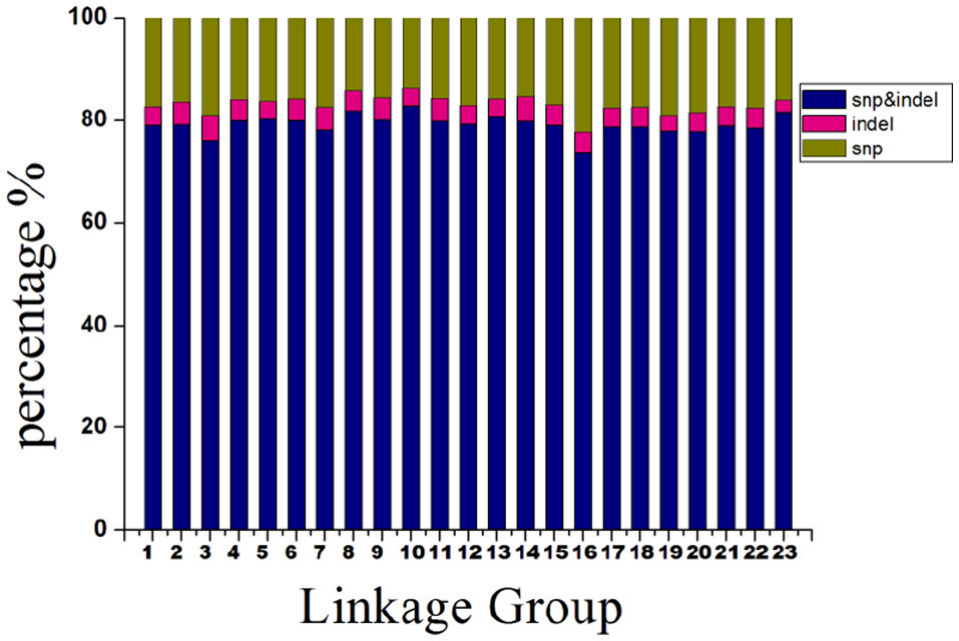
Percentages of different types of SLAFs on each linkage group.

The 14,189 SLAFs on the integrated map were composed of three types of markers. These were 11,228 “SNP-only,” 559 “InDel-only,” and 2,402 “SNP & InDel” types, with percentages of 79.13, 3.94, and 16.93%, respectively. The marker distribution of the three types on the 23 LGs are shown in Figure 4. LG10 had the largest percentage of “SNP-only” markers (82.82%) and the smallest percentage of “SNP & InDel” (13.87%), LG3 had the highest percentage of “InDel-only” markers (4.90%). A total of 23,664 SNP loci were further detected from the 11,228 “SNP-only” SLAFs and 2402 from the “SNP & InDel” SLAFs on the integrated map. Table 4 showed a different proportion of SNP types. The majority (61.68%) of the SNP markers was transition-type SNPs: 30.21% R (G/A) and 31.47% Y (T/C). The transversion-type SNPs (38.32%) consisted of S (G/C), M (A/C), K (G/T), and W (A/T) with the percentages between 6.17 and 13.60%.

**Table 4:** Statistic of six types of mapped SNPs.

**Type**	**Number**	**Ratio (%)**
W (A/T)	3,219	13.60
R (A/G)	7,148	30.21
M (A/C)	2,180	9.21
K (G/T)	2,189	9.25
Y (C/T)	7,447	31.50
S (C/G)	1,461	6.17
Total	23,664	100.00

### Evaluation of the genetic map

Heat map and haplotype map were used to estimate the map quality obtained in this work. The genetic map was based on multi-point recombination analysis. The closer the markers were, the smaller the recombination rate was. The heat map showed the recombination relationship between markers (Figure S6). The haplotype map reflects the double exchange and the genotyping of all the F1 individuals and parental controls. Graphical genotypes were generated based on the 14,189 SLAF markers. On the haplotype map, color change indicates the occurrence of a recombination event (Figure S7). A high percentage of the recombination blocks were defined by their color difference. Most of the LGs performed well according to the Figures S6, S7.

## Discussion

Genetic linkage maps are useful tools for genomic studies. They can elucidate the genome structure and facilitate the identification of molecular markers pertinent to biological traits in an experimental segregating progeny. It is particularly important to construct maps for long generation species, such as tree species. This study has developed the first reference genetic linkage map for *O. fragrans*, a species for which there is relatively little genomic information. This could be a useful starting point for further genetic studies on this plant.

### Sample population

Establishing a suitable population is the first step in constructing a genetic map. As a long-lived and woody tree, it is very difficult to obtain the traditional mapping materials for *O. fragrans*, such as Double Haploid (DH), Recombination Inbred Lines (RILs) and F2 progeny, which have been widely used to construct the maps for many different crops. However, *O. fragrans* is a highly heterozygous species where the F1 hybrids often show substantial and different types of segregation (Wu et al., 2010). The pseudo-testcross strategy provides a simple way to undertake genetic mapping (Grattapaglia and Sederoff, 1994), and has been successfully applied to the varity of tree species, such as *Malus pumila* (Maliepaard et al., 1998), *Anacardium occidentale* (Cavalcanti and Wilkinson, 2007), *Eucommia ulmoides* (Wang et al., 2014), and *Populus* sp. (Cervera et al., 2001; Yin et al., 2004). Sweet osmanthus is a functional androdioecy (Hao et al., 2011). A controlled pollination was performed between “Huangchuan Jingui” (hermaphrodite) and “Wanyin Gui” (male). Then, the hybrid seeds were in vitro embryo cultured to circumvent the long and hazardous germination period caused by the existence of a hard seed coat and after-ripening. Usually, the seeds of this species would germinate after stratification for 1 year, which completes the after-ripening period and breaks dormancy (Yang and Zhu, 2000). However, seeds rot very easily during this long pre-germination period. In this study, the F1 population with 129 offspring was established in just 2 months with a seedling rate of 90%, which provided a mapping resource and ensured that the map could be successfully constructed.

The size of the mapping population determines the saturation degree of the genetic map (Zamir and Tadmor, 1986). The sample population should be as large as possible to improve mapping accuracy. The experimental workload and cost, however, typically confine the size of the F1 population, and the existing maps of tree species are generally based on 100–150 individuals or even less (Gao et al., 2007). In this study, 129 F1 progenies were used to construct the *O. fragrans* genetic map. The key features of the map suggested that the population size was large enough to accurately develop the first framework map.

### The feasibility and advantages of the SLAF-seq technique

Conventional genetic mapping methods usually employ AFLP, RAPD, and SSR markers, especially for species where there is not enough genomic information (Wang et al., 1997; Brondani et al., 1998; Costa et al., 2000). Generally speaking, it is laborious and time-consuming to develop these markers for constructing maps using traditional experimental methods, particularly for a high-density linkage maps that need thousands of markers. The SLAF-seq method is a newly developed sequencing technique, which uses a simplified genome that has the specific loci needed for sequencing along with some restriction sites. Therefore, SLAF-seq can provide high numbers of accurate markers, and plays an important role in genetic analysis.

In this study, SLAF-seq yielded 173,537 markers from 206.92 M reads, and the polymorphic markers were successfully genotyped and sorted into five segregation types. After linkage analysis and filtering out the unsuitable SLAFs, 14,189 SLAF markers were mapped onto the LGs with a sequencing depth of 69.9-fold for parents and over 10-fold for progenies on average. The sequence depth showed a high degree of accuracy for the SLAF markers used in this research. In addition, 23,664 SNPs were developed from the mapped SLAFs that were sequence-tagged markers with co-dominant inheritance. These can be used for genetic diversity studies, germplasm identification, and comparative genomic studies on *Osmanthus*. This study suggests that the SLAF-seq method is a useful tool for developing molecular marker and constructing genetic map.

### Characteristics of the genetic linkage map

In this study, two comprehensive maps for the male and female progenitors have been constructed firstly, and then a integrated map consisting of a combination of the data from both progenitors was generated. The integrated map is 2962.46 cM in length with an average distance of 0.21 cM between adjacent markers. The average distance is considerably shorter than in the maps of other related species, such as *Olea europaea* (2n = 46) (De la Rosa et al., 2003; Charafi et al., 2007; Aabidine et al., 2010; İpek et al., 2016). The maximum gap larger than 5 cM was found in the LG7, LG9, and LG18, which may represent the regions lacking marker polymorphism, a failure to detect markers, or hot spots for recombination (Lindahl, 1991; Zhang et al., 2011; Wang et al., 2012). The number of linkage groups was 23, which was equal to the haploid chromosome for *O. fragrans* (Li et al., 2004). The length of 23 linkage groups was arranged in descending order, which was significantly correlated (*r*^2^ = 0.882, *p* < 0.01) with the size of chromosomes reported by Li et al. (2004). This data may also indicate the quality of the map constructed in this study. Once some markers or genes are located on a certain chromosome, the linkage groups could be accurately matched to the chromosomes. Table 2 showed that there were considerable differences in the number of markers present and the length of each LG. Furthermore, there was a strong correlation between the LG length and the marker numbers (*r*^2^ = 0.846, *P* < 0.01). The similar positive correlation was also detected in female and male maps (*r*^2^ = 0.885, *r*^2^ = 0.636, respectively, *P* < 0.01), so the different number of markers on the LGs would cause the difference length of the same LGs in both parent maps. LG8 showed the most difference in length and the order of some markers between the male and female maps, which may be caused because the relative positions of tightly linked markers are usually uncertain due to the effects of missing values and to differences in segregation information among markers (Maliepaard et al., 1997; Silfverberg-Dilworth et al., 2006).

Segregation distortion is a ubiquitous phenomenon in many species, which skews the frequency of alleles from the expected Mendelian ratio (Faris et al., 1998; Li et al., 2010, 2011). This phenomenon may be related to biological and environmental factors, such as a small mapping population size (Plomion et al., 1995), chromosome loss (Bradshaw and Stettler, 1994), or gametic and zygotic selection (Liebhard et al., 2003). The segregation distortion markers was uniformly distributed on the LGs, and no distorted markers were observed on LG8, LG16, LG19, and LG21. The information of the allele frequencies before and after fertilization is lacking. It is difficult to infer the segregation distortion due to the pre-zygotic selection or post-zygotic selection. However, about 20% seedlings died within a month before sampling without other conditions, which suggested that some post-zygotic selection mechanism may have played an important role in the segregation distortion ratios of this study. “Wan Yingui” and “Huangchuan Jingui” owned a far genetic relationship (Shang et al., 2004), diverging strongly in many important morphological traits, such as gender and corolla color, which were chosen as parents for hybridization, and may lead to segregation distortion more easily than the parents with close genetic relationship (Kianian and Quiros, 1992; Xian-Liang et al., 2006). Some reports have shown that segregation distortion markers can increase the LG genetic length of the map and improve QTL mapping when the segregation distortion was properly incorporated (Luo et al., 2005; Xu, 2008; Zhang et al., 2010). 950 skewed markers (*P* < 0.05) were added to the final map, which should improve any future QTL genotype mapping.

It is the first reference map that was reported in this study for *O. fragrans*. No phenotypic traits were located on the present map because the progenies in our study were still seedlings, and no traits of ornamental or agronomic interest could be segregated and evaluated because of the juvenile nature of the seedlings. The mapping population resources have been planted at the Garden of Huangchuan Jingui for several months where the climate is suitable for the growth of this species. Once the progenies become sexually mature, this high-density linkage map could offer a useful platform for future QTL mapping, including the important traits of flowering time, corolla color, flower fragrance, and cold resistance. The markers on this map were developed at level of the whole genome. Therefore, the constructed map also serves as an important reference for gene cloning and genome structure investigations into *O. fragrans*.

## Author contributions

FS and WY designed and organized the entire project. YxH, WY, MD, and YjH performed the experiments. YxH analyzed the data and drafted the manuscript, and FS revised it. All authors read and approved the final manuscript.

### Conflict of interest statement

The authors declare that the research was conducted in the absence of any commercial or financial relationships that could be construed as a potential conflict of interest. The reviewer PG and handling Editor declared their shared affiliation

## Supplementary material

Figure S1 Linkage group 1 for Huangchuan Jingui (female), Wan Yingui (male) and their integration.

Figure S2 Linkage group 2 to 7 for Huangchuan Jingui (female), Wan Yingui (male) and their integration.

Figure S3 Linkage group 8–14 for Huangchuan Jingui (female), Wan Yingui (male) and their integration.

Figure S4 Linkage group 15–19 for Huangchuan Jingui (female), Wan Yingui (male) and their integration.

Figure S5 Linkage group 20–23 for Huangchuan Jingui (female), Wan Yingui (male) and their integration.

Figure S6 Heat map of the integrated maps.

Figure S7 Haplotype map of the integrated maps.

Table S1 Information of the marker on the19 linkage groups (LGs).
