# Supplementary figures and images for: The First Genetic Map in Sweet Osmanthus (Osmanthus fragrans Lour.) Using Specific Locus Amplified Fragment Sequencing
Source: Front Plant Sci. 2017 Sep 22;8:1621. doi: 10.3389/fpls.2017.01621 (PMC5614988; doi:10.3389/fpls.2017.01621)

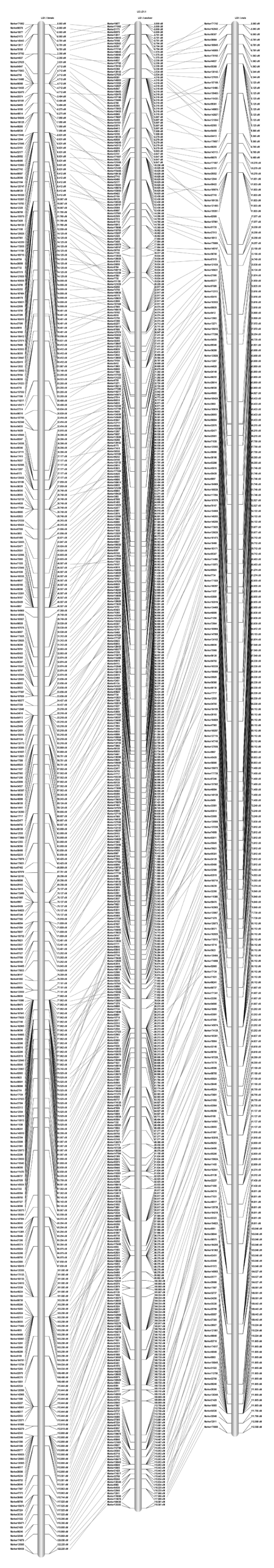

Supplement: Figure S1 — Linkage group 1 for Huangchuan Jingui (female), Wan Yingui (male) and their integration. [file Image1.JPEG]

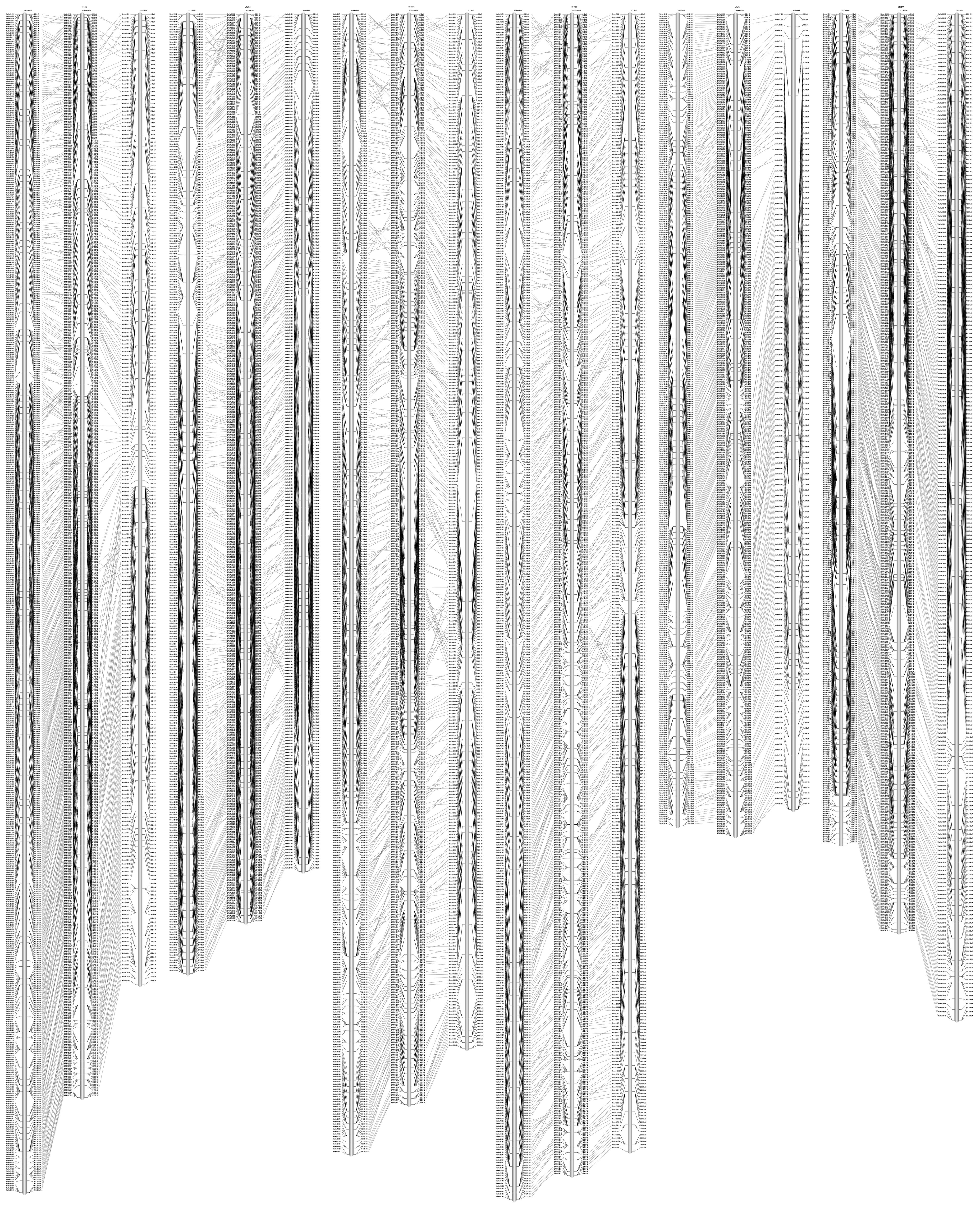

Supplement: Figure S2 — Linkage group 2 to 7 for Huangchuan Jingui (female), Wan Yingui (male) and their integration. [file Image2.JPEG]

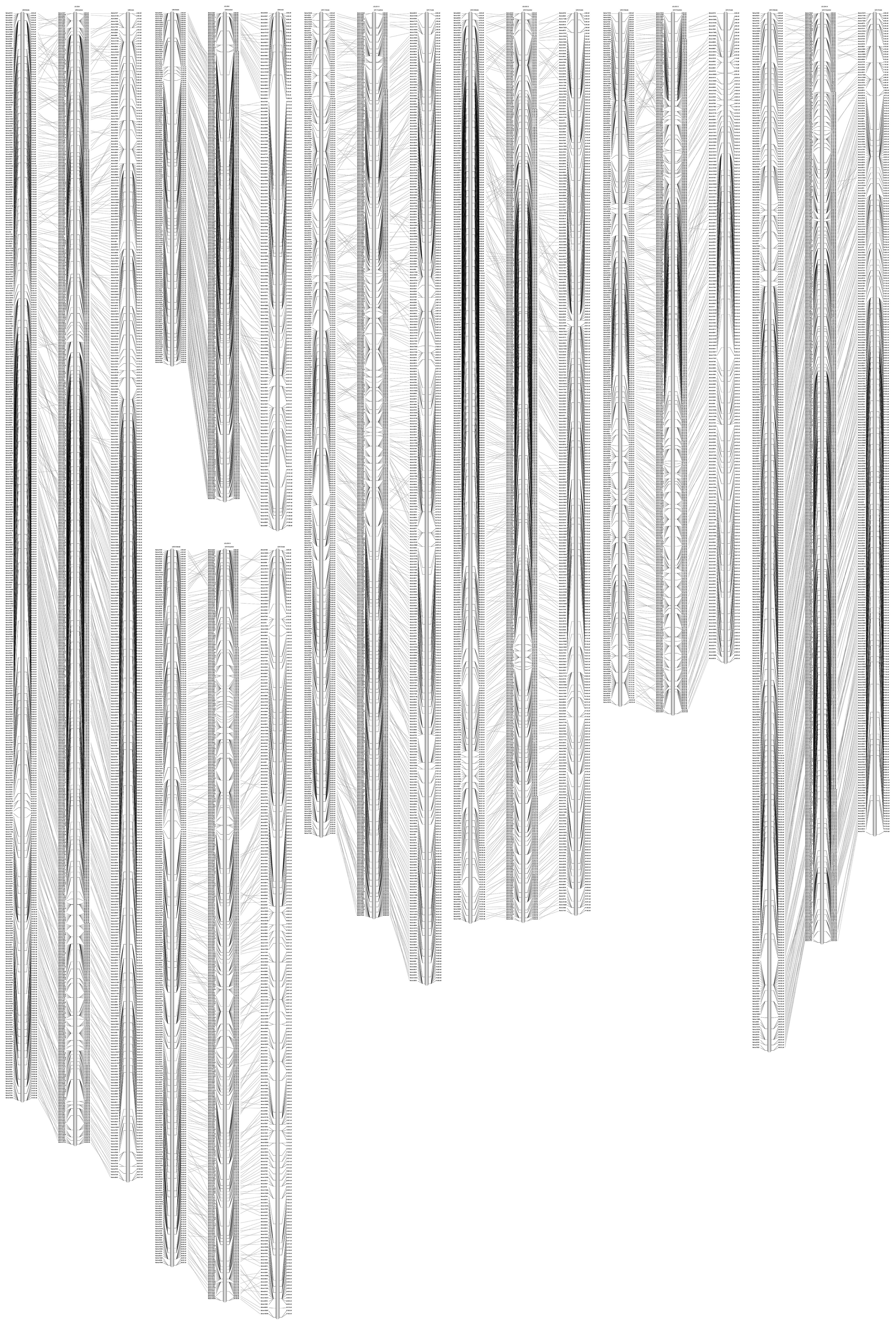

Supplement: Figure S3 — Linkage group 8–14 for Huangchuan Jingui (female), Wan Yingui (male) and their integration. [file Image3.JPEG]

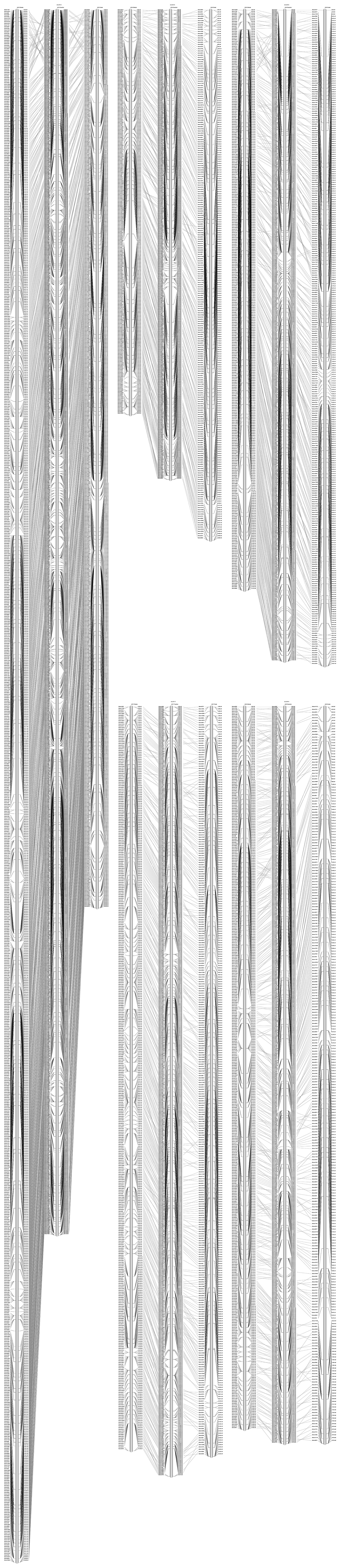

Supplement: Figure S4 — Linkage group 15–19 for Huangchuan Jingui (female), Wan Yingui (male) and their integration. [file Image4.JPEG]

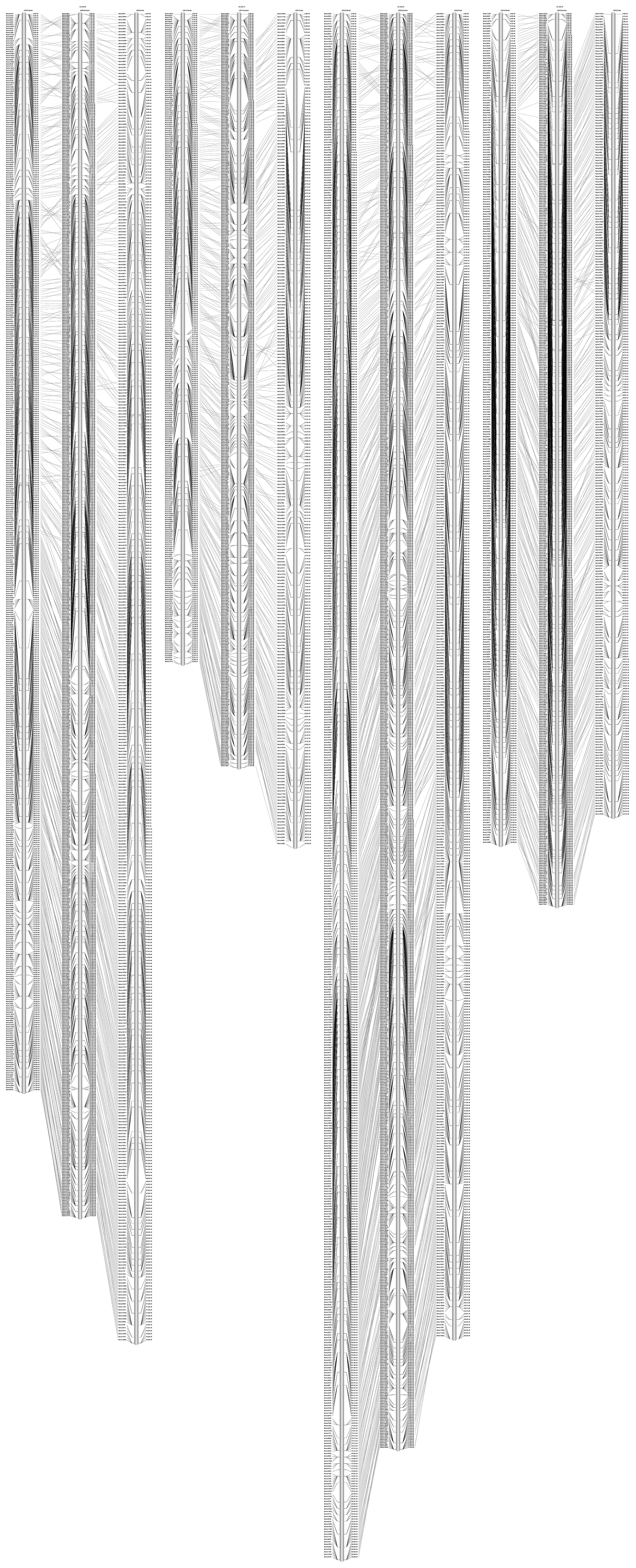

Supplement: Figure S5 — Linkage group 20–23 for Huangchuan Jingui (female), Wan Yingui (male) and their integration. [file Image5.JPEG]

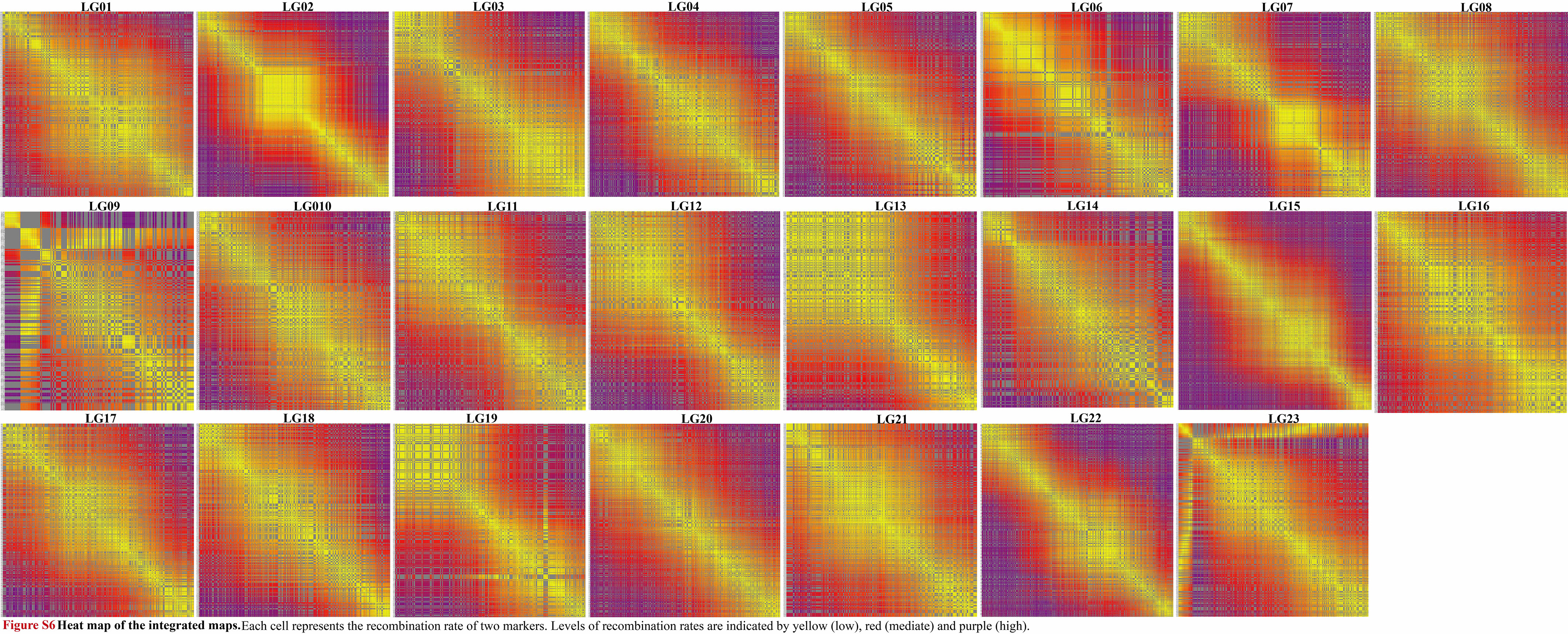

Supplement: Figure S6 — Heat map of the integrated maps. [file Image6.JPEG]

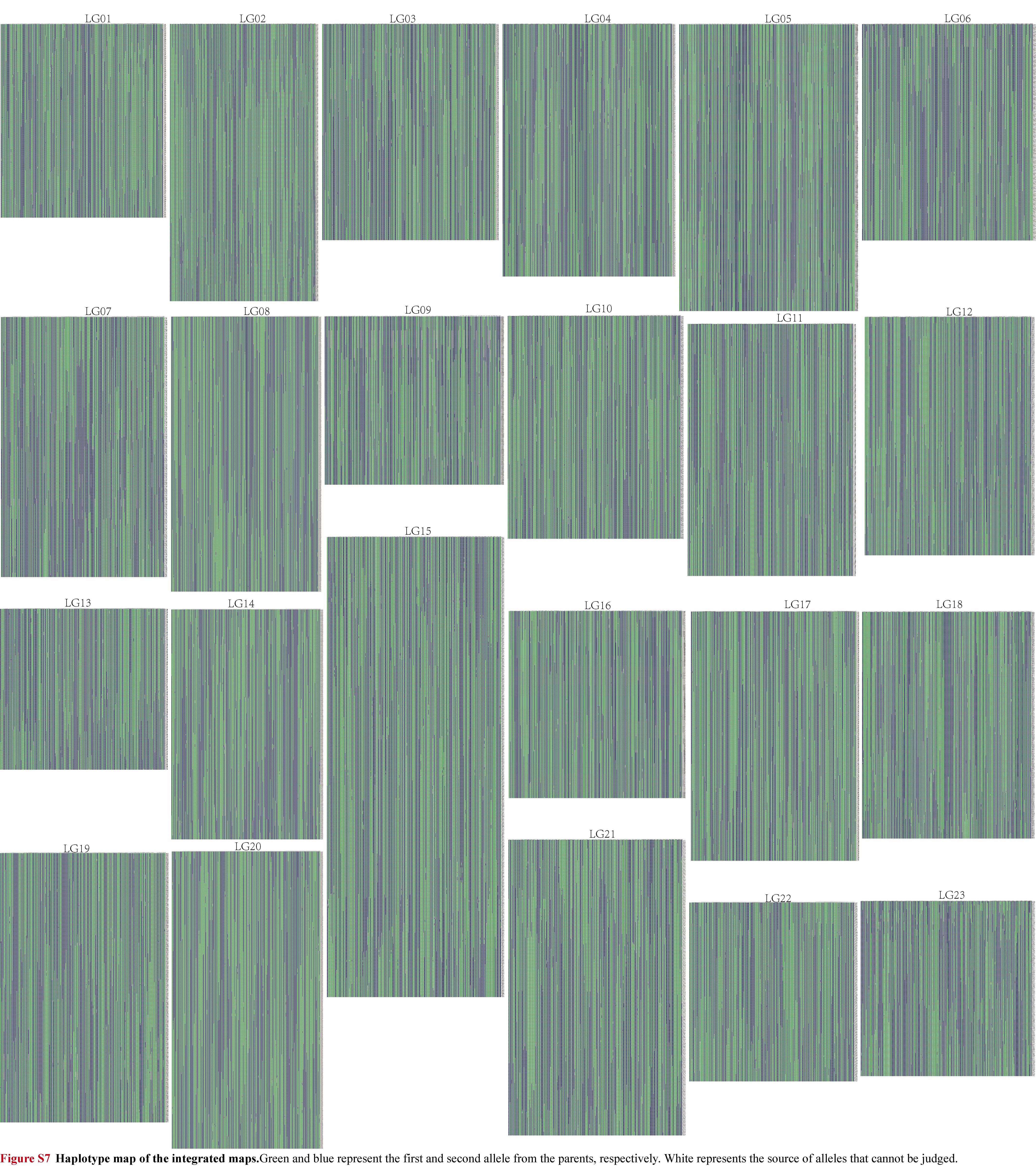

Supplement: Figure S7 — Haplotype map of the integrated maps. [file Image7.JPEG]
